# Investigation of the association between foot-and-mouth disease clinical signs and abattoir serological data in large ruminants in northern Lao People’s Democratic Republic

**DOI:** 10.3389/fvets.2024.1392885

**Published:** 2024-07-29

**Authors:** Emily Gee, James R. Young, Syseng Khounsy, Phouvong Phommachanh, Peter Christensen, Watthana Theppangna, Tom Hughes, Tom Brownlie, Adisone Temmerath, Alex Inthavong, Phoummavanh Inthapanya, Sivone Punyasith, Stuart D. Blacksell, Michael P. Ward

**Affiliations:** ^1^Sydney School of Veterinary Science, The University of Sydney, Camden, NSW, Australia; ^2^Mahidol-Oxford Tropical Medicine Research Unit, Faculty of Tropical Medicine, Mahidol University, Bangkok, Thailand; ^3^National Animal Health Laboratory, Vientiane, Lao People's Democratic Republic; ^4^Conservation Medicine, Sungai Buloh, Selangor, Malaysia; ^5^Ingenum Limited, Dunedin, New Zealand; ^6^Nuffield Department of Medicine, Centre for Tropical Medicine and Global Health, University of Oxford, Oxford, United Kingdom

**Keywords:** FMD, clinical signs, outbreak predictors, surveillance, Lao

## Abstract

Foot-and-mouth disease (FMD) is a highly infectious and endemic disease in Lao PDR. However, surveillance is weak, and outbreaks are not routinely reported. To address this, serum samples were routinely collected from cattle and buffalo from provincial abattoirs between November 2021 and December 2022. A total of 2,663 serum samples were collected from large ruminants (*n* = 1,625 cattle; *n* = 1,038 buffalo) from 17 provinces. Samples were tested for specific antibodies directed against FMD non-structural protein (NSP) to determine the proportion of animals exposed to FMD virus. In addition to sampling from abattoirs, further independent data was collected to report clinical signs and outcomes from 94 districts in 12 northern provinces. These incident reports were recorded by district staff using a Google Form and summarised monthly in the National Animal Disease Reporting System. Information was collected on species, incident date, herd size, location and which clinical signs the animals presented. Overall, 46% of the tested animals returned a positive result using ID Screen® FMD NSP Competition ELISA. Results from serological testing were then compared with reported clinical signs from the same district. In districts reporting ‘mouth problems’ (regardless of other clinical signs) the median FMD seroprevalence was 49.7%, compared to 31.6% in districts not reporting mouth problems (*p* = 0.021). This finding suggests that reporting clinical cases of ‘mouth problems’ could be a potential predictor of FMD infection at a district level in cattle and buffalo in Lao PDR. Furthermore, in districts reporting ‘fever’, ‘mouth problems’, and ‘nose/mouth secretions’ together, the median FMD seroprevalence was 46.2%, compared to 24.4% in districts not reporting these signs (*p* = 0.033). In districts reporting ‘mouth problems’ and ‘nose/mouth secretions’ the median FMD seroprevalence was 49.4%, compared to 25.5% in districts not reporting these signs (*p* = 0.037). In districts reporting both ‘fever’ and ‘mouth problems,’ the median FMD seroprevalence was 46.4% compared to 25% in districts not reporting these signs (*p* = 0.017). Based on serological data generated by abattoir surveillance, this study identified clinical signs most predictive of FMD seroprevalence. These novel findings can be used to guide passive surveillance efforts in the future specifically in northern Laos and help support improved FMD surveillance more broadly in FMD endemic countries in Southeast Asia.

## 1 Introduction

Foot-and-mouth disease (FMD) is a highly infectious disease that is endemic in the Lao People’s Democratic Republic (Lao PDR or Laos) (1). FMD affects cloven-hoofed animals such as cattle, sheep, goats, pigs, and other wild species (2). This disease is caused by the foot-and-mouth disease virus (FMDV; genus *Apththovirus*, family *Picornaviridae*). It has a low infectious dose and the virus can spread quickly among susceptible animal populations (3). Inhalation of the virus, direct and indirect contact with infected animals, and exposure to contaminated fomites are the primary modes of transmission (3). Pathogenicity of FMDV is species specific and influenced by host dynamics; for example, pigs produce relatively large amounts of aerosolised FMDV, while cattle shed less virus but are more susceptible to infection (4).

Foot-and-mouth disease is an important transboundary disease that causes substantial production losses and consequently leads to international trade disruption (5). FMD has major negative economic impacts on countries in the region (6, 7). Financial losses have previously been determined, including direct losses due to mortality (100% of pre-FMD sale value) and morbidity (difference between the expected sale price pre-FMD and 1 month following onset of FMD), and indirect losses due to costs of treatments (6). The losses due to FMD per household varied between provinces (*p* < 0.001) and were USD 1,124, USD 862 and USD 381 in Luangprabang, Xiengkhuang, and Xayaboury, respectively, being 60, 40, and 16% of annual household income (6). Comparison of the costs of FMD with annual household income from sales of large ruminants indicated losses of 213, 181, and 60% of the income in LPB, XK, and XYL, respectively (6). The variation in losses between provinces was due to differences in levels of morbidity with highest in LPB, treatment methods with antibiotic use common in LPB, age of animals sold and sale prices with higher prices in XK (6). Similarly, studies conducted in Cambodia provide a similar picture of FMD impacts at the household level (7). Countries infected with FMD cannot trade live animals with FMD free countries, and trade of livestock products is also restricted (8). Lack of access to lucrative markets has further consequences; it restricts the development of commercial farming, further exacerbating poverty alleviation (8).

FMD continues to threaten food security and public health in Laos and other low- and middle-income countries (LMIC), where a significant proportion of the population’s primary income is dependent on agriculture (9). In Laos, there are approximately 1.9 million cattle, 1.2 million buffaloes, 3.1 million pigs, and 0.5 million goats (10), and FMD is present in buffalo, cattle (10), and pigs (11). In a recent study, a seroprevalence of 44.6% in cattle and 35.0% in buffalo was reported from abattoirs in six provinces between March and December 2019 (10). Data collected between 2012 and 2016 in Southern Laos revealed a FMD seroprevalence of more than 50% in adult ruminants (older than 5 years) while an active.

Survey in Xiengkhouang (XKG) province in 2017 demonstrated 33.2% seroprevalence (12). Early incursion detection and effective tracing of animals, herds, and locations that may have been exposed are essential for effectively combating FMD outbreaks (3). The primary challenge for regional FMD control is that Laos shares borders with five other countries where the disease is endemic, and is located on a major path for transboundary animal movements within the Greater Mekong Subregion (13). Other obstacles also exist, such as the high buffalo population compared to neighbouring countries and low vaccination rates (14). In Laos, FMD control is difficult due to frequent movement of animals, lack of resources, general awareness, poor biosecurity, inadequate diagnostic and vaccine technology, and financial constraints (15). FMD likely contributes to smallholder poverty through not only production losses but also through the financial burden of both the treatment of ill animals and control measures such as vaccination (9, 13). To improve surveillance in Laos, efforts were initiated in 2018 by the Department of Livestock and Fisheries (DLF) and the National Animal Health Laboratory (NAHL) within the Ministry of Agriculture and Forestry (MAF), in collaboration with Mahidol Oxford Tropical Medicine Research Unit (MORU) (16). A surveillance programme was developed aiming to utilise the existing network of District and Provincial Agriculture and Forestry Officers (DAFO & PAFO) to collect and report FMD information and collect and submit livestock serum samples from provincial abattoirs to be used for surveillance (16).

Outbreak investigation, laboratory diagnosis and reporting remain constrained in Laos, primarily due to lack of capacity and resources (16, 17). Cost effective solutions are needed to improve reporting of high-impact animal diseases (16, 17). Furthermore, there is strong regional demand for beef for human consumption, and Lao PDR would like to participate in the trade, but the lack of information about FMD is a constraint on participation (16).

This study aims to identify which clinical signs are most strongly associated with FMD in cattle and buffalo based on serological sampling at abattoirs and observations of clinical signs reported by DAFOs & PAFOs. The findings of this study could be applied to guide cost-effective surveillance activities in the future.

## 2 Materials and methods

### 2.1 Project timeframe and organisation contributions

The Laos Cambodia Thailand 4 One-Health and transboundary disease project (LACATH4) commenced on July 1st, 2021, and ran for 24 months through to June 30th, 2023. This project was the fourth phase of a research surveillance programme, primarily investigating serum samples from abattoirs, and was a collaborative effort implemented by the Department of Livestock and Fisheries (DLF), National Animal Health Laboratory (NAHL) and Mahidol Oxford Tropical Medicine Research Unit (MORU) (16, 17). The design of the current study was driven by the field data available.

### 2.2 Study area and target population

Reporting of clinical signs was completed in 12 Northern Provinces of Laos: Bokeo, Borikhamxay, Huaphanh, Luangnamtha, Luangprabang, Oudomxay, Phongsaly, Vientiane Capital (CT), Vientiane Province (PV), Xayaboury, Xaysomboon, and Xiengkhuang. Within these provinces, 92 individual districts provided information on observed clinical signs of large ruminants (cattle and buffalo) that were the target population for this study. Serum samples were collected from both cattle (*n* = 1,625) and buffalo (*n* = 1,038) from abattoirs within 86 districts from 17 provinces in Laos: Bokeo, Borikhamxay, Champasack, Huaphanh, Khammuane, Luangnamtha, Luangprabang, Oudomxay, Phongsaly, Saravane, Savannakhet, Sekong, Vientiane Capital (CT), Vientiane Province (PV), Xayaboury, Xaysomboon, and Xiengkhuang. Cattle and buffalo sampled at abattoirs were not the same animals from which clinical signs were observed and reported.

### 2.3 Eligibility criteria

Exclusions to the primary dataset were applied to make the abattoir and clinical signs reporting data comparable. Specifically, for a district to be included, both abattoir and clinical signs had to be reported. As a result, provinces without matching districts were excluded from this study to ensure that both datasets could be validly compared. No other exclusions were applied to the data.

### 2.4 Sample collection and transport

A total of 2,663 large ruminant serum samples were collected by trained PAFO staff between November 2021 and December 2022. With the agreement of PAFO prior to the study commencement, at each collection, up to 10 cattle and 10 buffalo samples were obtained (if available) using convenience sampling selection. Other than species, no further sampling criteria was performed. Sample collection was performed within the last 10 days of every month, and samples were dispatched to the Veterinary One Health Reference Laboratory (VOHRL) located within the National Animal Health Laboratory (NAHL) in Vientiane. Samples were generally collected from live animals at the abattoirs and, on rare occasions, and if animals were not yet at the abattoir, on farms prior to animals being transported to the abattoir. Samples were collected from the jugular or coccygeal vein. The amount of blood collected varied depending on the size of the animal, with the maximum amount being 10 mL of blood. The alternate collection method was done at slaughter or immediately after (within minutes). For this method, blood was collected from a stream coming from the neck and caught in an open, needle-capped syringe.

To collect the serum, blood (in a syringe or vacutainer) was placed at a 45° angle on a rack away from any direct sunlight and heat. This sat at room temperature for up to 2 h to allow the blood to clot. If, after 2 h, the serum had not yet separated from the clot, the blood was placed in the refrigerator at approximately 4°C for around 16 h. Once the serum had separated, the serum was poured into a tube labelled with the batch and sample ID. The serum samples were then placed into a plastic bag labelled with the batch ID. Serum samples could then be stored at 4°C for up to 10 days; if long-term storage was required, they were stored at −20°C. Serum samples were packed using a triple packing technique to avoid breakage or leakage of the sample containers. Once packed, the samples were shipped to the VOHRL located within the NAHL.

### 2.5 Laboratory testing

Laboratory testing was performed by veterinary staff at the VOHRL located within the NAHL. Serum samples were tested for antibodies against FMD non-structural protein (NSP) using the ID Screen FMD NSP Competition ELISA (18). This test allows for differentiation between infected and vaccinated animals (14). The ELISA was performed according to the manufacturer’s instructions in the kit. Based on the manufacturer’s procedure, S/N%, less than or equal to 50%, was considered a positive result (S/N% ≤ 50% = positive), and greater than 50% was considered negative (S/N% ≥ = negative). Monthly result reports were also approved for release by the NAHL Director and distributed to DLF, provincial governors, PAFO staff, and the FAO.

### 2.6 Clinical reporting

Following notification from and consultation with farmers, DAFOs and PAFOs completed the reporting of clinical signs, which was independent from the abattoir surveillance described above (2.4). The National Animal Disease Reporting System (NADRS) uses a Google form to collect information from each district, including information on the species, time and date, province, and which clinical signs were observed. The clinical signs available for selection within the Google form were: Lethargic, Fever, Anorexic, Lameness, Diarrhea, Bleeding, Cough, Sneeze, Seizure, Skin lesion, Swollen leg, Swollen face, Eye problem, Mouth problem, Vomit, Dehydrate, Pale, Cyanosis, Generalised Rash/Redness, Localised Rash/Redness, Nose/Mouth Secretion, Hypothermia, Asymptomatic, and other symptom. One District Agriculture and Forestry Officer (DAFO) per district reported this information, stored online and shared between NAHL, DLF and LACATH4. This data was then reviewed every 1–3 days to identify potential outbreaks, and a monthly summary report was produced. Monthly reports were approved for release by NAHL Director and distributed to DLF, provincial governors, PAFO staff, and the Food and Agriculture Organization of the United Nations (FAO) Emergency Centre for Transboundary Animal Diseases in Laos.

### 2.7 Analysis

Data was entered into a Microsoft Excel™ spreadsheet. For each province and each district included in the study, seroprevalence was estimated, and the reported clinical signs were summed and the data were matched based on unique province-district identity. Temporal matching of data was not attempted. As the data sources of abattoir samples and clinics signs are independent, i.e., not from the same animals, reported clinical signs did not directly correspond to serum samples taken. To test for any spatial association between the datasets, the comparison was made between seroprevalence from samples collected at abattoirs and clinical signs (or a combination of clinical signs of ‘fever,’ ‘mouth problems,’ ‘nose/mouth secretion,’ which are consistent with known FMD clinical signs) reported by PAFOs in the same district. Districts were also classified as reporting versus not reporting the various clinical signs, regardless of other clinical signs, and the difference in seroprevalence was tested using a median test in SPSS (IBM Statistics SPSS V28). A map of seroprevalence was prepared by importing district-level seroprevalence and clinical signs data into a GIS (ArcGIS v 10.5. ESRI) and joining with a district-level shapefile of Laos (DIVA-GIS).^1^ Geographic Coordinate System WGS 1984. Choropleth and proportional symbol maps were then created to visualise the distribution of FMD seroprevalence and the sum of clinical signs reported. Clustering was tested using Moran’s spatial autocorrelation (Spatial Statistics. ESRI), using the sum of reported clinical signs (e.g., number of reports of mouth problems) as the outcome variable at the district level.

## 3 Results

### 3.1 Eligible data included in analysis

Of all the serum samples (*n* = 2,663) collected, 1,225 samples returned a positive result using the ID Screen FMD NSP Competition ELISA (18) (apparent prevalence of 46%). Province-level seroprevalence is summarised in Table 1 (prior to excluding districts from which clinical signs data was not reported). Seroprevalence ranged from 20.2 (Phongsaly) to 75% (Xaysomboon). The median seroprevalence was 50.9%. After the exclusions were applied to create an analysis dataset in which only districts from which seroprevalence and clinical signs were included, 1,100 serum samples from 43 districts in 10 northern Laos provinces were included in the final analysis.

**Table 1:** FMD seroprevalence in 10 northern provinces in Lao PDR (*n* = 1,394).

Province	Sum of positive samples	Total samples	Prevalence (%)
Bokeo	116	216	53.7
Borikhamxay	102	198	51.5
Huaphanh	42	147	28.6
Luangnamtha	4	15	26.7
Luangprabang	66	162	40.7
Oudomxay	77	148	52.0
Phongsaly	36	178	20.2
Vientiane^*^	22	50	44.0
Xaysomboon	33	44	75.0
Xiengkhuang	120	236	50.8

Serum samples were collected from cattle and buffalo from November 2021 to December 2022 via abattoir sampling.

^*^Vientiane CT (17/20/85.0) and Vientiane PV (5/30/16.7) data combined.

### 3.2 Description of the dataset

#### 3.2.1 Abattoir data

Four hundred eighty-four of the 1,100 samples returned a positive result using the ID Screen FMD NSP Competition ELISA (18) (apparent prevalence of 44%).

**Figure 1:**
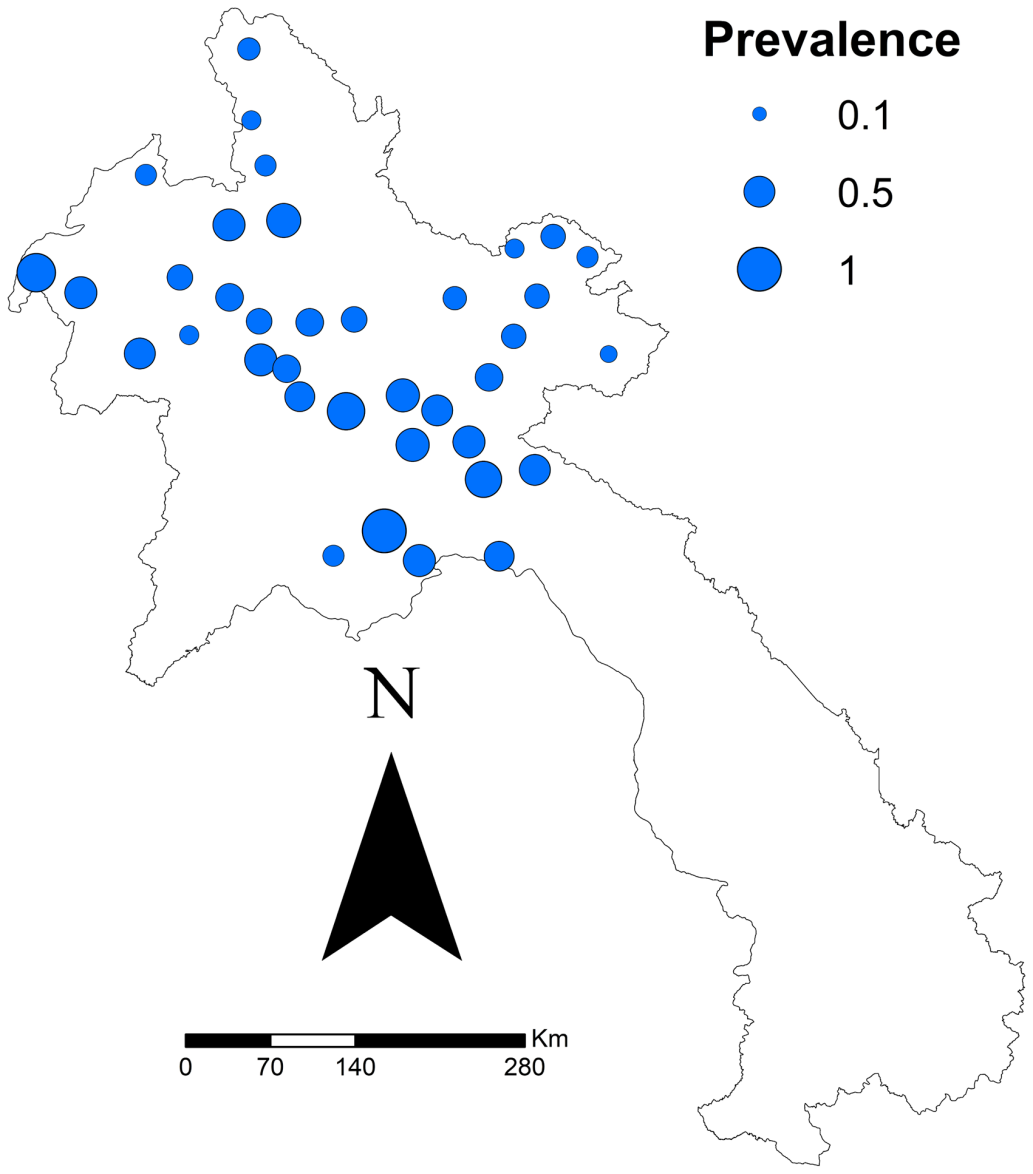
District-specific seroprevalence estimated (number positive ÷ total tested) in a study of FMD cattle and buffalo in 10 provinces in northern Laos from November 2021.

Figure 1 shows a map of districts proportional to seroprevalence. District seroprevalence ranged from 0% (Meung, Bolikhanh, Long, and Thoulakhom) to 100% (Long Chaeng). The median seroprevalence was 50%. The autocorrelation analysis identified no clustering overall for seroprevalence (Moran’s autocorrelation Index = −0.0121; *p* value = 0.9189).

#### 3.2.2 Google form data

**Figure 2:**
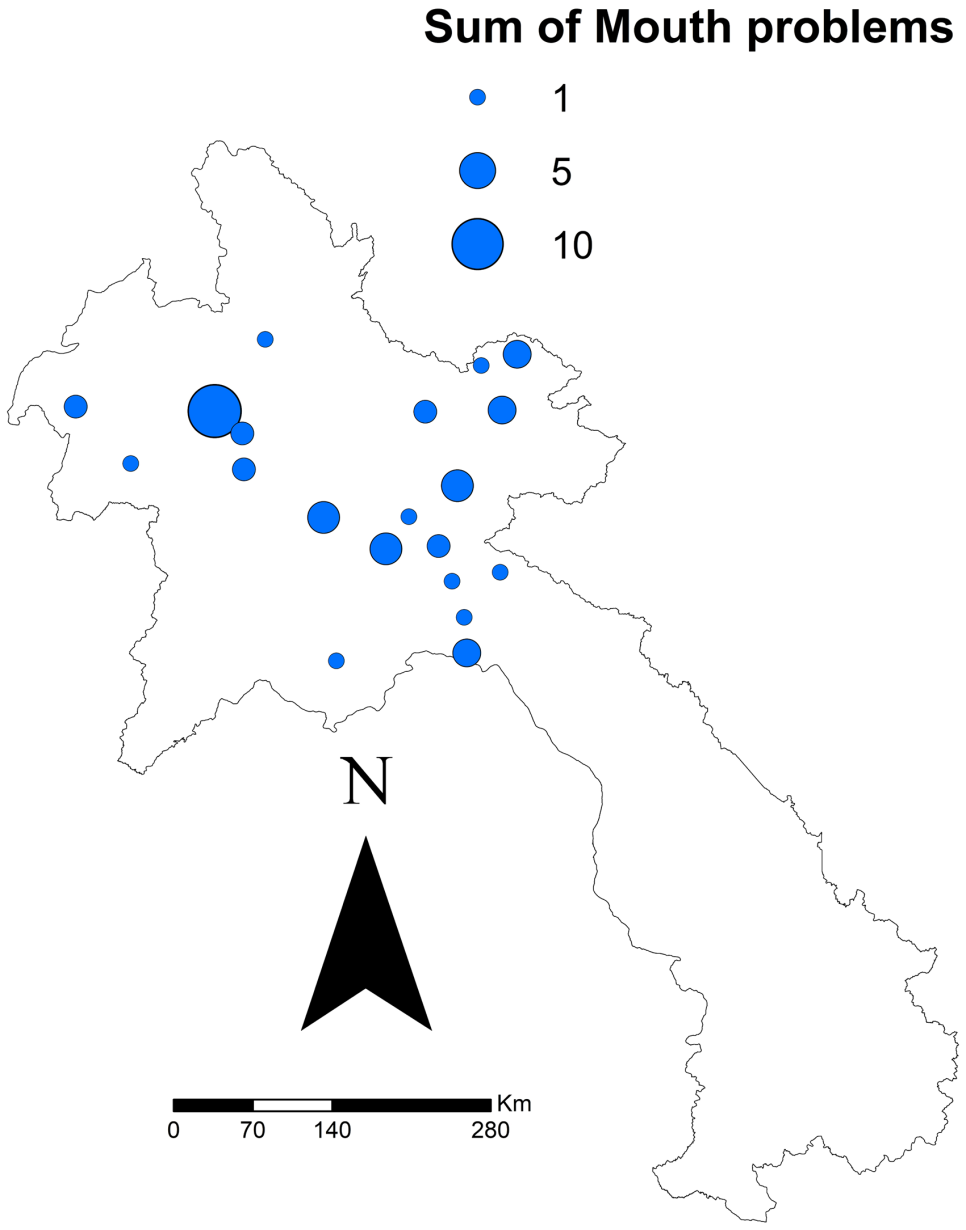
Sum of ‘mouth problems’ reported by 43 district in 10 northern Laos provinces in a study of FMD in cattle and buffalo from November 2021 to December 2022. Data was reported via the National Animal Disease Reporting System.

**Figure 3:**
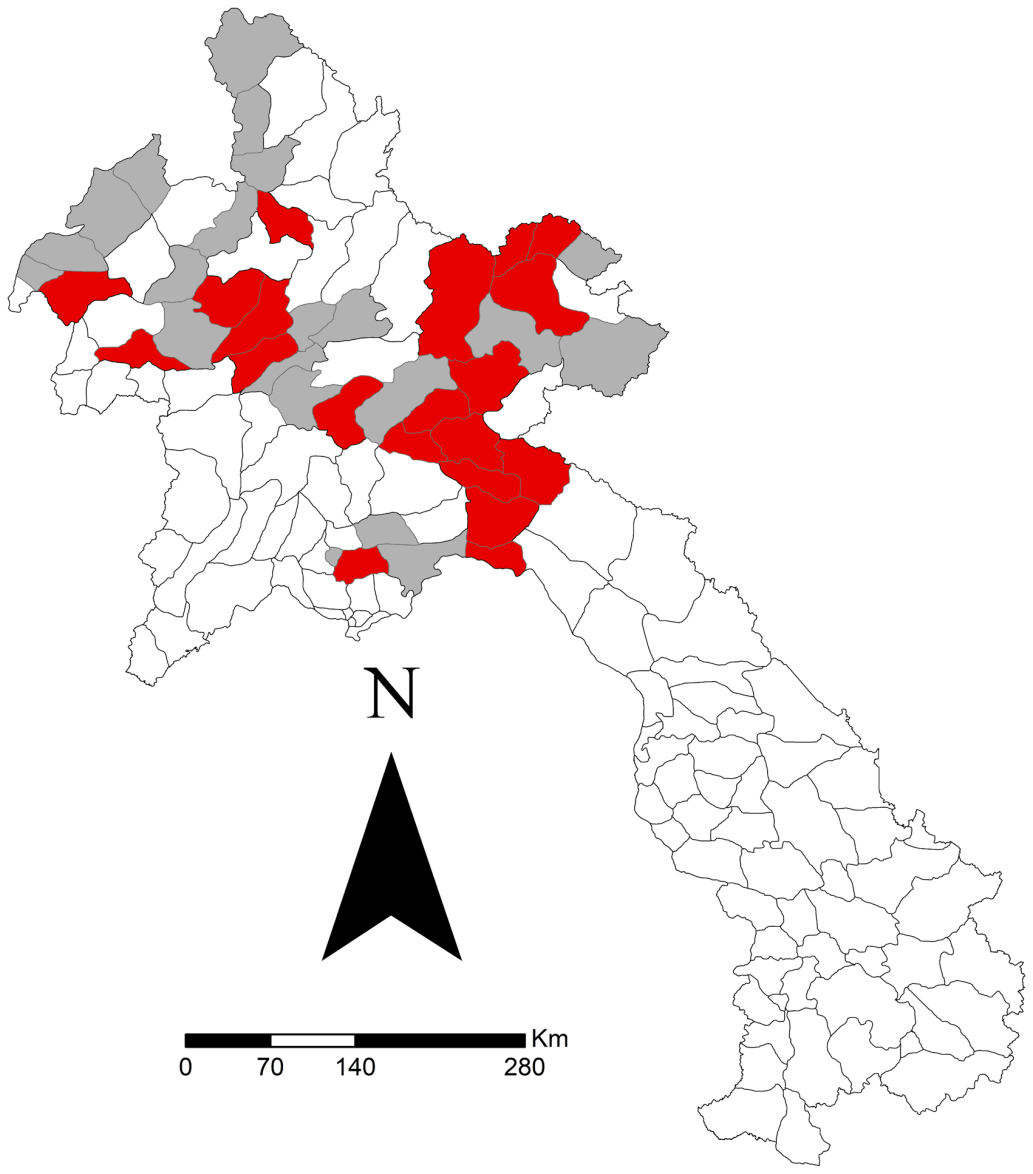
Districts reporting ‘mouth problems’ (red) versus those not reporting (grey) in a study of FMD in cattle and buffalo in 43 districts in 10 northern Laos provinces from November 2021 to December 2022. Data was reported via the National Animal Disease Reporting System.

After all exclusions, 667 reports from 43 districts were analysed. The specific sums of each clinical sign reported are outlined in Table 2. There was a significant difference for one clinical sign: ‘mouth problems’ (*p* = 0.021). Regardless of other clinical signs, the median seroprevalence in districts reporting ‘mouth problems’ was 49.7%, compared to 31.6% in districts not reporting ‘mouth problems.’ Figure 2 shows the sum of all reported ‘mouth problems’ at the district level. Figure 3 shows those districts reporting ‘mouth problems’ and those not reporting this clinical sign. When assessing multiple clinical signs (‘fever,’ ‘mouth problems,’ ‘nose/mouth secretion’) and their association with FMD seroprevalence, all syndromes showed a similar association. In districts reporting ‘fever,’ ‘mouth problems,’ and ‘nose/mouth secretions’ together, the median FMD seroprevalence was 46.2%, compared to 24.4% in districts not reporting these signs (*p* = 0.033). In districts reporting ‘mouth problems’ and ‘nose/mouth secretions,’ the median FMD seroprevalence was 49.4%, compared to 25.5% in districts not reporting these signs (*p* = 0.037). In districts reporting both ‘fever’ and ‘mouth problems,’ the median FMD seroprevalence was 46.4% compared to 25% in districts not reporting these signs (*p* = 0.017). The autocorrelation analysis identified no significant dispersion for the sum of ‘mouth problems’ (Morgans Index = −0.0625; *p* value = 0.7233).

**Table 2:** Number of reports for each clinical sign reported in 10 northern provinces and 43 districts of Laos in a study of FMD in cattle and buffalo from November 2021 to December 2022.

Clinical sign reported	Number of reports	Number of provinces	Number of districts
Lethargy	124	9	29
Fever	84	8	26
Anorexic	67	8	18
Skin lesion	61	4	4
Lameness	55	8	21
Mouth problem	52	8	22
Secretion from nose or mouth	41	6	15
Swollen leg	32	8	26
Diarrhoea	30	8	24
Generalised rash/redness	25	9	18
Asymptomatic	17	7	10
Eye problem	8	4	6
Seizure	6	2	2
Cyanosis	5	3	3
Localised rash/redness	5	4	5
Bleeding	4	8	19
Pale	3	3	3
Sneeze	2	1	1
Cough	1	3	3
Dehydrated	1	1	1
Vomiting	0	0	0

The National Animal Disease Reporting System uses a Google form to collect information from each district on observed clinical signs.

## 4 Discussion

This study found that districts reporting that cattle and buffalo with clinical signs of ‘mouth problems,’ or ‘mouth problems’ together with ‘fever’ and/or ‘nose/mouth secretions,’ have a higher FMD seroprevalence compared to districts not reporting these clinical signs in cattle and buffalo. Additionally, the three identified syndromes all show similar associations with FMD seroprevalence: ‘fever’ and ‘mouth problems’ and ‘nose/mouth secretions’ (*p* = 0.033), ‘fever’ and ‘mouth problems’ (*p* = 0.017), and ‘nose/mouth secretion’ and ‘mouth problems’ (*p* = 0.037). These findings suggest that these clinical signs could be potential predictors of FMD infection at a district level in cattle and buffalo in Laos.

FMD affects a wide range of cloven-hoofed species, and clinical signs vary not only between species but also between different age groups and breeds. In addition, the viral strain and an animal’s previous exposure to the virus and vaccination status will impact the signs with which the animal will present (19). Despite this, there are a few classical signs seen in most cases. These include fever and vesicular lesions in the mouth, which eventually rupture and slough off, leaving erosions and excessive drooling (19). In cattle, the erosions are generally observed on the muzzle, in the mouth, and in and around the nostrils (19). In this study mouth problems, fever, and nose and mouth secretions (or a combination of these) were the clinical signs most significantly associated with animals testing FMD positive within the same district. Observation and reporting of these clinical signs could be used to predict FMD in Laos districts.

Due to the host species’ widespread lack of immunity, FMD infection typically presents with classical clinical signs in FMD-free areas. However, there has been increasing evidence that FMD infection in endemic areas could have more mild signs of disease (20). Previous studies have found that clinical outbreaks do not always provide an accurate picture of the amount of virus in the environment because subclinical infection can occur, particularly in immune cattle (21). Numerous studies have described how in livestock populations with a high level of immunity against FMD, where the disease has reached endemic stability or where vaccination programmes have been implemented, typical clinical signs we associate with FMD are suppressed (22, 23). Another study found that the proportion of FMD ELISA-positive animals was higher than those judged clinically positive (24). Many questions still exist regarding the different subclinical infection states and how FMD manifests in herds that are endemically infected (25). Findings from these studies highlight the need to consider subclinical infections in the overall control and prevention strategies for FMD. Additionally, understanding the role of asymptomatic animals in the spread of the disease can help to guide and improve surveillance efforts and enhance early detection measures in the future (20). The seasonal variation in disease expression also requires further investigation.

One limitation of this study was the sample size due to the necessary exclusions. Only 1,100 out of the total 2,663 serological samples and 667 of the total 1,071 clinical reports were used in the final analysis. With more districts included, future research could include predictive modelling of FMD seroprevalence based on reported clinical signs to better establish the usefulness of this form of surveillance in the southeast Asian context. Furthermore, the available data was only collected over a 13-month period, which may not have been sufficient to capture all FMD outbreaks accurately. The reporting strategies may introduce measuring bias, as those reporting clinical signs are trained PAFOs, not qualified veterinarians. This bias may result in over-reporting the more apparent signs but under-reporting of those that may be more subtle. Clinical signs selected for reporting may need to be descriptive enough for unqualified staff to report. This is unlikely to be a major issue regarding syndromic surveillance as it is focused more on identifying trends, not a valid diagnosis of FMD. An ecological study design was used based on the availability of surveillance data. Such a study design has limitations when used to infer cause and effect. However, when used at the district level this approach can provide options for authorities to increase the efficiency of surveillance by reducing or avoiding the need for laboratory-based surveillance in a resource-limited setting. Study results should be interpreted as predictive, not inferential. Finally, there were limitations associated with using Google Forms for data collection; although it is cost-effective, it poses challenges regarding its maintenance and potential quality issues. Further research that expands the data collection timeframe and use of molecular diagnostics to confirm clinical FMDV would help manage any potential sampling bias. A review of the clinical signs listed in the Google Form may also assist in improving capturing more specific outbreak data.

The findings of this study give insight into the current reporting behaviours of FMD in Northern Laos, as well as highlight recent seroprevalence data and clinical signs most likely to be associated with disease. This information could allow for improved passive surveillance, therefore allowing for early detection and implementation of control measures. Surveillance is often limited and insufficient in endemic areas such as Laos, relying predominantly on passive outbreak reporting (20), and almost no active surveillance is performed due to lack of resources.

Passive surveillance often results in delays in observing and reporting key clinical signs without incentivisation. The strong association between district-level observations and FMD seroprevalence reported in this study offers an opportunity to incentivise this passive surveillance. Addressing reporting constraints by supporting PAFO staff roles with emerging artificial intelligence offers a prescient option to increase the reporting capacity both in completeness and frequency of surveillance. Large language models can be trained to create comprehensive reports with relatively simple prompts and textual outcomes can be analysed in near- real-time alongside environmental datasets to drive greater local engagement. These outcomes can support decision support tools to differentiate subclinical infection states crucial for accurately assessing the seroprevalence of FMD in endemic areas.

The higher seroprevalence in districts reporting clinical signs of ‘mouth problems’ suggests that monitoring and early detection of the clinical sign of ‘mouth problems’ in animals could be an important factor in early identification and subsequently implementing control actions to assist in preventing the spread of FMD. These findings highlight the need to regularly monitor and detect mouth problems early. Identifying districts in which more animals display this clinical sign could allow for targeted surveillance and intervention strategies to be implemented, reducing the spread of the disease and minimising its impact on livestock populations. Furthermore, including additional clinical signs (‘mouth problems,’ ‘nose/mouth secretions,’ and ‘fever’) as a syndrome might provide even earlier detection of FMD.

Based on the findings of this study, syndromic surveillance paired with abattoir serological surveillance could be a novel valuable passive surveillance approach. Syndromic surveillance involves monitoring multiple clinical signs associated with disease to detect cases early, allowing for prompt intervention. Educating farmers, abattoir staff and PAFO officers to better understand and identify these clinical signs could help with FMD management and control. However, further studies are needed to validate the effectiveness of syndromic surveillance and refine its predictive capabilities. Whilst passive syndromic surveillance can be helpful, its benefits must be defined and measured.

## Data availability statement

The raw data supporting the conclusions of this article will be made available by the authors, without undue reservation.

## Ethics statement

The animal studies were approved by the Ministry of Agriculture and Forestry, Department of Livestock and Fisheries, Lao PDR; approval number 0019/DLF. The studies were conducted in accordance with the local legislation and institutional requirements. Written informed consent was obtained from the owners for the participation of their animals in this study.

## Author contributions

EG: Conceptualization, Investigation, Methodology, Software, Writing – original draft, Writing – review & editing. JY: Conceptualization, Investigation, Project administration, Supervision, Writing – review & editing. SK: Investigation, Project administration, Resources, Supervision, Writing – review & editing. PP: Investigation, Methodology, Project administration, Validation, Writing – review & editing. PC: Data curation, Investigation, Methodology, Writing – review & editing. WT: Data curation, Investigation, Project administration, Writing – review & editing. TH: Project administration, Writing – review & editing. TB: Methodology, Validation, Writing – review & editing. AT: Data curation, Methodology, Writing – review & editing. AI: Data curation, Investigation, Methodology, Writing – review & editing. PI: Investigation, Methodology, Writing – review & editing. SP: Investigation, Methodology, Writing – review & editing. SB: Funding acquisition, Investigation, Methodology, Project administration, Resources, Supervision, Writing – review & editing. MW: Software, Supervision, Writing – original draft, Writing – review & editing, Formal analysis, Methodology, Project administration, Resources.

## Footnotes

^1^ diva-gis.org
